# Trial of Goule for Murder

**Published:** 1845-10

**Authors:** 


*Trial of Goule for murder:—Plea of Insanity.* (*Northern Circuit.*)—Henry Louis Goule was indicted for the murder of his wife.

It appeared that the prisoner and his wife lived very unhappily together, in consequence of jealousy on the part of the former. He accused her of undue intimacy with different gentlemen.

On the 10th of June, the prisoner reached home about seven o'clock. After sitting for a short time, he rose hurriedly, buttoned his coat, and went out, at half-past seven. He was absent about an hour and a half. When he returned, he grinned at Mrs. Goule, who was then at the inside, and he at the outside, of the window. He then went in. He appeared a little flurried; but became calmer. Mrs. Goule sat at the window, in such a position as to see every one coming up the street on the opposite side. When the prisoner came in, he sat opposite Mrs. Goule: it was about half-past nine. He said to her sister-in-law, "Jane, do you think Emma innocent?" She replied, that she did. The deceased said, "You hurt me; you wound my pride, Henry; you doubt my virtue." At this she rose up. At that time there were two gentlemen coming up the street, on the opposite side. She said, "There is Mr. Scruton and Mr. Jepson, I will call them in and have this cleared up." He had often named Mr. Scruton to his wife, and to her sister-in-law. He said that Scruton was too familiar with his wife; and this he said several times. It was a frequent topic of conversation. The deceased went towards the door: the prisoner stood before her, and said,—"Don't." She said,—"I will." She then went to the door, which was ajar; and so did the prisoner, who attempted to stop her. The prisoner put his hand behind him, and pulled a pistol out of his right-hand pocket. He said, "Then, if you will, take this;" and he fired. She had put up her left arm between her and the pistol. The ball hit her left arm. She opened the front door, screaming out, "Oh God! I am shot." She crossed the street: the prisoner followed: without stopping, he took aim with another pistol at his wife. He fired, when running after her. A surgeon was called in; but on the 17th she died of lock-jaw.

The following is the substance of the exculpatory evidence:—

Mary Jane Gile, a relation of the prisoner, the principal witness for the prosecution, stated,—"Within the last fortnight the defendant complained very much of his head; I have often heard him say, he thought a wound in his head affected him."

Mr. Stoke, an assistant to a surgeon, said,—"Mr. Goule was wounded in the turmoil of the pitmen some time ago; it was a very serious wound; I was called in, when he was seized with a fit; since he received this wound, I have frequently seen him in an excited state."

Mr. Green, surgeon, said,—"The wound sustained by the prisoner on the occasion referred to by the last witness was of a very severe character. Before it was perfectly healed, he wandered away, and remained for some days. When he came back, he was in a very excited state. He has frequently been very wild in his eye, and eccentric in his manner since. It frequently happens, that persons who have had concussion of the brain are excited at intervals, and lose all control over themselves. When the prisoner was in his senses, he was a very superior man."

Verdict, "Not Guilty, on the ground of Insanity."—*Ibid.*

